# A random graph model of density thresholds in swarming cells

**DOI:** 10.1111/jcmm.12757

**Published:** 2016-02-19

**Authors:** Siddhartha G. Jena

**Affiliations:** ^1^ Harvard CollegeCambridgeMAUSA

**Keywords:** aquaporin‐1, membrane composition, water transport, cholesterol

## Abstract

Swarming behaviour is a type of bacterial motility that has been found to be dependent on reaching a local density threshold of cells. With this in mind, the process through which cell‐to‐cell interactions develop and how an assembly of cells reaches collective motility becomes increasingly important to understand. Additionally, populations of cells and organisms have been modelled through graphs to draw insightful conclusions about population dynamics on a spatial level. In the present study, we make use of analogous random graph structures to model the formation of large chain subgraphs, representing interactions between multiple cells, as a random graph Markov process. Using numerical simulations and analytical results on how quickly paths of certain lengths are reached in a random graph process, metrics for intercellular interaction dynamics at the swarm layer that may be experimentally evaluated are proposed.

## Introduction

Swarming behaviour refers to collective movement in a population of organisms and has been found to occur in individual cells, herds of cattle and flocks of birds 1, 2; it is best described as a group of organisms moving purely through individual directives. Swarming can occur on two or three dimensions; however, for the purpose of this study, we will focus on populations of single cells, moving through intercellular interactions. A textbook example of this cell–cell interaction is swarming in bacterial colonies 3. This process, in comparison to tissue formation and bird flocking, occurs largely on the two‐dimensional plane of the media on which the cells grow, and much data have been collected on swarming and continue to be collected, as bacterial motility and its governing forces are of great interest in the laboratory and clinic. *Proteus mirabilis*, a gram‐negative bacterium, displays swarming behaviour and has been demonstrated to form multicellular rafts of elongated and hyperflagellated swarmer cells 4. This differentiation into the swarmer phenotype has been found to precede the swarming motility of the entire population of cells. Due partly to a large consensus that an understanding of swarming in bacteria could hold the key to understanding collective biological behaviour in larger cellular ensembles, a great number of mathematical models of swarming have arisen in recent years. However, since swarming comprises so many individual cellular and population‐level behaviours, these mathematical models differ significantly in how effectively they are able to explain certain behaviours. For example, the stepwise process of swarming has been modelled as a series of differential equations 5, 6. This model faithfully recreates the concentric ‘swarm rings’ that are the trademark of swarming behaviour, but tells us very little about the underlying dynamics occurring at each swarm front. However, this is not due to a lack of adequate modelling strategies; surprisingly little is known about these dynamics on the experimental level. Intercellular interactions are incredibly important and are required for a wide range of biological phenomena to occur. In some cases, particularly in bacterial swarming motility, these phenomena are dependent on local cell density 7; the compounded effect of numerous identical interactions occurring within a subset of the population. In this study, we offer an explanation of bacterial swarming using graph theoretical methods.

If we represent the bacterial cells as discrete ‘vertices’ and the interactions between them as ‘edges’ or lines connecting these vertices, we have a simple framework for quantifying interaction‐related characteristics of a population. Using this *graph theoretical* framework, which we will rigorously define later in this article, we can not only quantify the number of interactions in a given subset of a population but also measure how these quantities change as the graph evolves over time.

To better motivate the current investigation, let us take a brief detour into physics, turning to the tools that are used to understand phase changes in condensed matter systems. Specifically, we refer to a model in condensed matter physics called the random cluster model, which makes extensive use of a mathematical object called a *random graph*. This is a graph defined as a set of vertices, with an additional parameter *p* incorporated which represents the probability that there exists an edge between any two vertices. This model is used extensively to represent interactions between particles on a dimensional lattice as a graph. Using random cluster models, materials such as metals, liquids and other ensembles of atoms or particles can be analysed to a surprising level of accuracy. This model can be applied to systems which are variations on the Ising model, in which particles are coupled on a lattice. Phase changes signify changes in states of matter and therefore in the underlying arrangement of particles in matter. In nature, these can be as simple and easy to observe as watching water freeze or ice melt; indeed, the phase diagrams for the more common materials have been in existence for quite some time. However, the atomic level interactions that lead to phase changes are a bit more subtle and hard to understand using conventional approaches. Yet, when placed under the lens of the relatively simple random cluster model, we find a great deal of connections between random graphs and measurable phenomena in physics. For example, many phase changes occur at a critical edge parameter of the random graph; in other words, when a certain level of connectivity 0 < p_c_ ≤ 1 is achieved, the system as a whole will undergo a phase change 8. For generalized systems, this critical point has been proven to be p_c_ = 0.5; however, in our model, we will derive a specific result for our bacterial system.

We apply the above theory to our model of threshold cell densities, as a direct parallel can be drawn from assemblies of particles in an amorphous solid or other highly correlated condensed state to assemblies of cells which have a threshold density. A unique property of these graphs is that they maintain an understanding of the interactions between individual particles, while yielding important measures of critical phenomena such as phase changes in the material. In the present study, we use random graph models, often used to give analytical results of phase changes in condensed matter systems, to demonstrate the dynamics through which local density thresholds are reached. We treat a given cellular density threshold as a ‘connected component’ of a fixed size and work through an analytical result for the time taken to reach such a connected component in a random graph process.

## Mathematical formalism

### Graphs

*Definition*: A *graph* is described as a collection of *vertices V* = {*v*_1_, *v*_2_, *v*_3_,…,*v*_*i*_} and *edges E* = {*e*_1_, *e*_2_, *e*_3_,…,*e*_*j*_} where *j* ≤ *i*, where an edge is simply a line drawn between two vertices. The presence of edges in a graph can be represented as a matrix with dimensions *i* × *i,* where the existence of an edge between vertices *v*_*n*_ and *v*_*m*_ is represented by a positive non‐zero value at (*m*,*n*), which signifies the weight of the edge 9.

*Definition*: A graph can be *weighted* in that some of the non‐zero values can be larger or smaller than others, which is useful for representing, for example, signalling networks where certain signals are stronger or more robust than others.

There are many measurable graph properties, but only several key measurable traits of a given graph *G*{*V*,* E*} that we will study in this article are discussed here. They are *graph isomorphisms, supergraphs, subgraphs and connectedness*.

*Definition*: Two graphs *G* = {*V*,* E*} and *G*′ = {*V*′, *E*′} are *isomorphic* if there exist a pair of functions *f*:* V* ➔ *V*′ and *g*:* E* ➔ *E*′ such that *f* associates each element in *V* with exactly one element in *V*′ and vice versa; *g* associates each element in *E* with exactly one element in *E*′ and vice versa, and for each *v* in *V*, and each *e* in *E*, if *v* is an endpoint of the edge *e*, then *f*(*v*) is an endpoint of the edge *g*(*e*) 10.

*Definition*: A graph *H*{*V*″, *E*″} is a *subgraph* of the graph *G* = {*V*,* E*} if *V*″ is a subset of *V* and *E*″ is a subset of *E*. *G* is, therefore, the *supergraph* of *H*.

*Definition*: Two nodes *v*_1_, *v*_2_ are *connected* if there exists an edge between them.

*Definition*: A graph *G* = {*V*,* E*} is *connected* if for any two *v*_*i*_, *v*_*j*_ in *V*,* E* is such that there exists a path between *v*_*i*_ and *v*_*j*_.

There are various different types of graphs, but as we are not dwelling on theory but rather exploring the applications of graphs to a topic in biology 11, it will be more enlightening if we cover these graphs as they naturally arise.

### Markov chains

*Definition*: A *Markov chain* is a sequence of random variables *X*_1_, *X*_2_,…, *X*_*n*_, that follow the property that the probability *P*(*X*_*n*_ = *x*_*n*_ ¦ *X*_1_ = *x*_1_, *X*_2_ = *x*_2_,…, *X*_*n*−1_ = *x*_*n*−1_) = *P*(*X*_*n*_ = *x*_*n*_ ¦ *X*_*n*−1_ = *x*_*n*−1_). The countable set *S* = {*X*_1_, *X*_2_, *X*_3_,…, *X*_*i*_} is called the *state space* of the Markov chain 12.

In a set *S* containing a certain number of states, called Markov states, we can imagine a Markov chain as ‘jumping’ from state to state, while following pre‐described probabilities for the transition between different states. What this means is that the process is non‐deterministic. The matrix corresponding to the Markov chain is, as we would expect, a *stochastic* matrix, which means that for each state that the chain is in, the sum of the probabilities of getting to any of the other states is 1. Now, even though these structures are referred to as ‘chains’ in much of the literature, this simply refers to a 1‐to‐1 Markov process. Any process, however, that describes moving between states *via* a stochastic matrix in which the probabilities are non‐deterministic can be described as a Markov process. Individual Markov states need not be quantities; rather, in this application, we are treating each state in the Markov process as a graph.

## Creating the model

Let us treat the time propagation of an assembly of cells as a random graph process, with individual cells represented as discrete vertices on the graph. Our objective is to find the time for the consolidation layer to form, given a certain interaction threshold *m*.

A *graph process* 11, 12 is a sequence of graphs on the same set of vertices, *V*. In our model, this means that the spatial arrangement of the vertices stays the same. We define the graph process with the definition from Bollobas 13:

- Each *G*_*t*_ is a graph on *V* the set of vertices
- *G*_*t*_ has *t* edges for *t* = 0, 1,…, *N*.
- *G*_0_ is a subset of *G*_1_ which is a subset of *G*_2_… is a subset of *G*_*t* max_.

**Figure 1:**
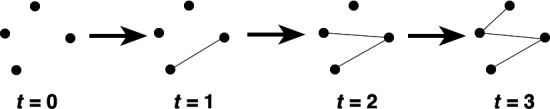
Random graph process on 4 vertices. Each transition has a probability of 1, so achieving a certain result, *i.e*. a path of connected cells, is controlled by the randomness of the graph.

In most biological phenomena, the system is under a certain amount of ‘pressure’ from its surroundings to respond to a given stimuli within a certain timescale. Therefore, in this particular application of this model, we examine graphs with a certain *t*_max_, so that each random graph *G*(*n*, α) can be defined by two indices: *N* the number of vertices in the graph and α the probability that any two randomly chosen vertices in the graph are connected at *t*_max_. We next define the *hitting time* τ of the given monotone property *Q*, a term of our own devising which is the time it takes to reach a certain property: *Τ* = min{*t* ≥ 0 such that *G*_*t*_ has *Q*}. These random graph processes can be treated as Markov chains, with the corresponding state space displayed in Figure 1 15, 16.

It has been found that swarming bacteria of multiple species undergo a phenotypical differentiation during their swarming phase that results in swarming cells being hyperelongated in addition to being grouped in ‘raft’ structures 17, 18, 19. These rafts consist largely of cells lying side by side, so that a rafting group of cells can be approximated as a network of cells that forms a long chain, with each cell connected to a maximum of two adjacent cells 20, 21. This simplifies our search for a connected component, since the limitation placed upon the degree of connectivity of each cell refines our search to a connected ‘chain’ subgraph. We can show, using the definition of random graphs, that this property is indeed monotone; because *G*_0_ is a subset of *G*_1_ which is a subset of *G*_2_… is a subset of *G*_*t* max_, the adjacencies that are present in *G*_*i*_ will carry over to *G*_*i*+1_ and so at any timestep the length of a given string of cells will either stay the same or will increase but will not decrease. We can utilize this particular property of random graph processes by assuming that the interactions leading up to the formation of a cell–cell raft component are stochastic but that once the component is formed it is permanent.

Having established the general parameters for our model and the biological assumptions that are being made in our calculations, we can approach the problem of reaching the cellular density threshold in several ways. These are described and explored here.

### Model 1

In our first, and most basic model, we consider the graph *G*_1_(*V*,* E*), abbreviated as *G*_1_, where the set of vertices *V* = *v*_1_, *v*_2_,…, *v*_*N*_. The graph process is then computed as follows:

1. At each timestep, two random numbers 1 ≤ *i* ≤ *N* and 1 ≤ *j* ≤ *N* are generated from a uniform distribution.
2. If *i* = *j*, nothing happens.
3. If an edge already exists between *v*_*i*_ and *v*_*j*_ nothing happens.
4. If an edge does not already exist between *v*_*i*_ and *v*_*j*_, an edge is created.
5. If either *v*_*i*_ or *v*_*j*_ is a node of degree 2 (the maximum degree of any node on our graph), nothing happens.

**Figure 2:**
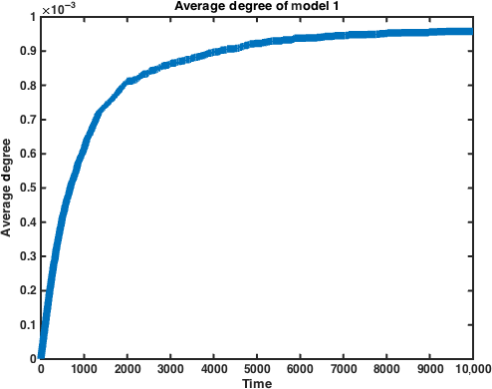
Average degree of nodes vs. time in Model 1. We can see that the average degree levels off as time approaches infinity. The average degree is measured over half of the adjacency matrix M_1_. Data represent average of *n* = 10 simulations.

**Figure 3:**
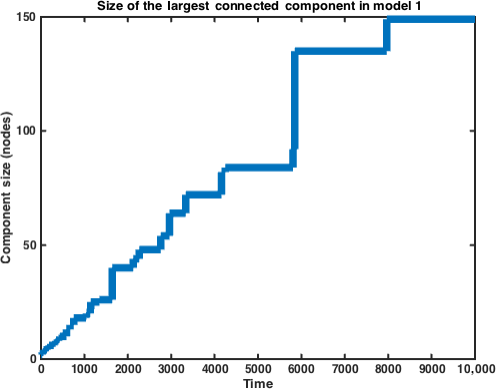
Size of the largest connected (giant) component vs. time in Model 1. Observations of random graph processes describe the ‘engulfing’ of smaller components by the giant component as time progresses, explaining the sudden increases in size of the giant component at *t* ≈ 1700 and 6000. Data represent average of *n* = 10 simulations.

This graph process can be compared to an assembly of cells faithfully forming a new interaction at almost every timestep but assumes that a maximum of only one interaction is formed at each timestep. It also excludes the biologically meaningless outcome of a cell connecting to itself. Regardless of its limitations, it provides a good starting model of how we imagine the swarming process to occur. The matrix representing this graph is an *N* × *N* square matrix, which we denote as the *adjacency matrix M*_1_, with *M*_1_(*i*,* j*) = 1 when *v*_*i*_ and *v*_*j*_ are interacting and *M*_1_(*i*,* j*) = 0 when *i* = *j* or if *v*_*i*_ and *v*_*j*_ do not interact. If we numerically simulate this process and measure the average degree of all of the nodes *v*_1_, *v*_2_,…, *v*_*N*_ over time for a graph with *N* = 1000, we have the figure in (Fig. 2):

As we can see, as *t* ➔ ∞, the average degree of *G*_1_(*t*) eventually becomes 2, our limit, as expected. The limit in the figure is taken over half of the adjacency matrix, which is symmetric over the diagonal. We can also measure the size of the largest connected component within our graph. We find that akin to descriptions of random graph processes, the giant component, once formed, ‘engulfs’ smaller connected components as time progresses, explaining the sudden increases in size that we see in our numerical simulation. Of course, in Model 1 if the simulation is allowed to continue, the size of the giant component approaches *N* (Fig. 3).

### Model 2

For our second model, we introduce a truly probabilistic element into our random graph process. Our algorithm for generating the graph process on *G*_2_ is similar to that for *G*_1_, with an added step. This new algorithm also allows for a realistic development of the swarming colony; in experimental conditions, the colony is not limited to one intercellular interaction per timestep. At each timestep, the following process loops over all pairs of vertices *v*_*i*_, *v*_*j*_ in *V*.

1. If *i* = *j*, nothing happens.
2. If an edge already exists between *v*_*i*_ and *v*_*j*_, nothing happens.
3. If either *v*_*i*_ or *v*_*j*_ is a node of degree 2 (the maximum degree of any node on our graph), nothing happens.
4. If an edge does not already exist between *v*_*i*_ and *v*_*j*_, an edge is created with probability *P*.

**Figure 4:**
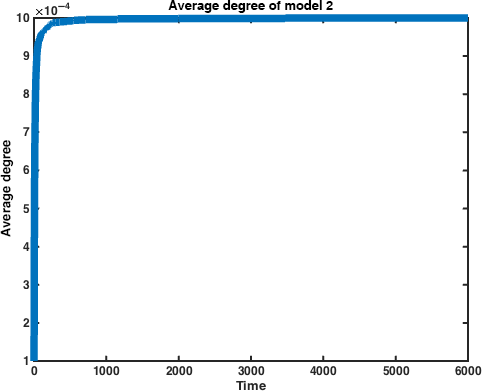
Average degree of nodes vs. time in Model 2. We can see that the average degree levels off as time approaches infinity. The average degree is measured over half of the adjacency matrix M_2_. Data represent average of *n* = 10 simulations.

Again, we examine the average degree of Model 2 for convergence. All numerical simulations were run with *N* = 1000 and with a probability of interaction *P* = 0.0001 (Fig. 4).

**Figure 5:**
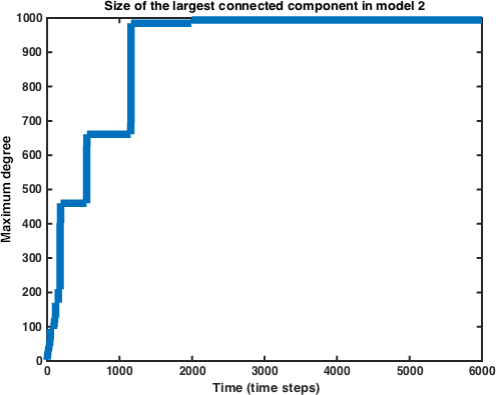
Size of the giant component vs. time in Model 2. Data represent average of *n* = 10 simulations.

As we did in Model 1, we track the size of the largest connected component as well. Model 2 converges on the giant component equalling the entire vertex space much more quickly than does Model 1. We next examine the role of the interaction probability *P* on the rate of this convergence. As we may expect, the rate of convergence decreases as the probability of any two cells interacting decreases (Fig. 5).

**Figure 6:**
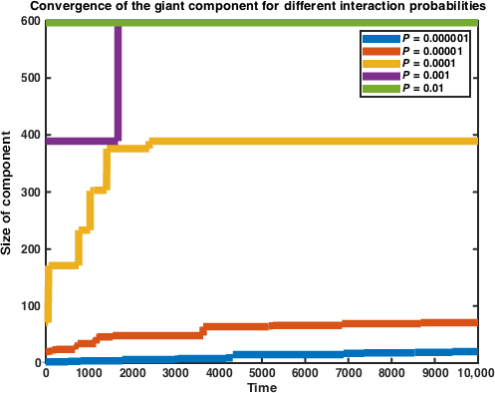
Size of the giant component vs. time in Model 2, for different values of the interaction probability *P*. We can see that as *P* approaches 1, convergence occurs at faster rates. Data represent average of *n* = 10 simulations.

**Figure 7:**
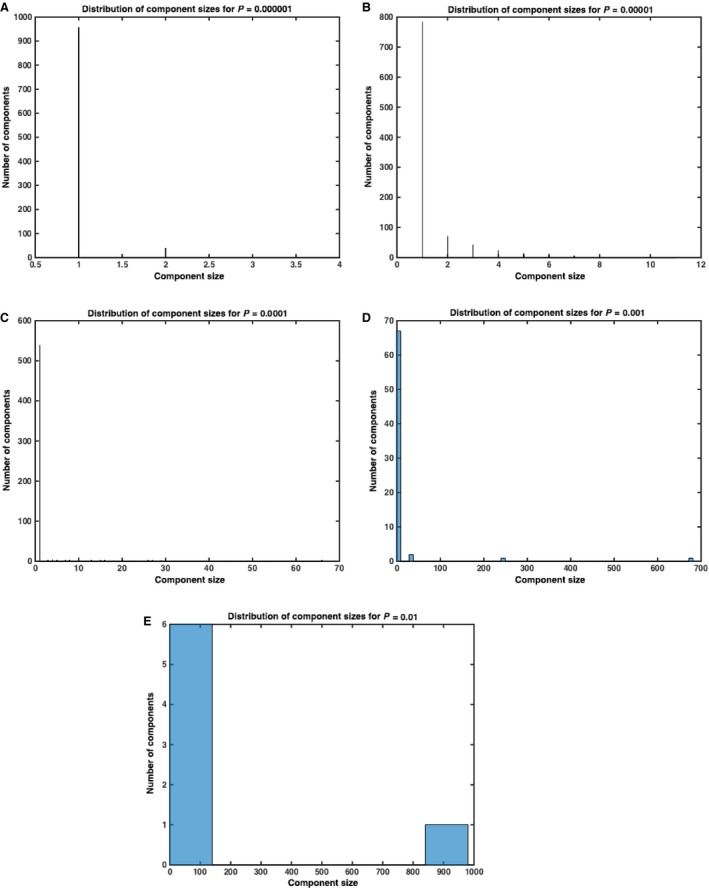
Distribution of component sizes for *P* = 0.000001, 0.00001, 0.0001, 0.001, 0.01, respectively, after 500 steps of numerical simulation under Model 2. As expected, the components are largely disparate when *P* ≈ 0 and the giant component gets larger as *P* approaches 1. Data represent average of *n* = 10 simulations.

Finally, we look at the distribution of connected component sizes at a fixed time. Mathematically, we can always extend our number of timesteps so that the size of the giant component approaches *N*. However, for biological systems, it is likely that there is some ideal time *t*_α_ ≪ ∞ after which the giant cell cluster is large enough that the population of cells begins to swarm. We, therefore, calculated the distribution of connected component sizes at time *t* = 500 for a population of size *N* = 1000. We can see that the distribution of component sizes is largely disparate when *P* ≈ 0 and the giant component gets larger as *P* ➔ 1 (Figs 6 and 7).

### Model 3

A characteristic element of cellular simulations is a birth–death process. This is an additional biological assumption that is often brought into use when considering populations of cells. However, the significance of the birth–death process changes when we are looking at interactions between cells in addition to scalar quantities representing connected components. Specifically, if a single cell dies, its connections to other cells are lost. We denote the probability of cell death to be *p*_*D*_ and construct a random graph process *G*_3_(*t*) with the following governing algorithm, which loops over all pairs of vertices *v*_*i*_ and *v*_*j*_ in *V*_3_.

1. If *i* = *j*, nothing happens.
2. If an edge already exists between *v*_*i*_ and *v*_*j*_, nothing happens.
3. If either *v*_*i*_ or *v*_*j*_ is a node of degree 2 (the maximum degree of any node on our graph), nothing happens.
4. If an edge does not already exist between *v*_*i*_ and *v*_*j*_, an edge is created with probability *P*.
5. Again looping over all cells in *V*_3_, a cell *v*_*k*_ in *V*_3_ has a probability *p*_*D*_ of ‘dying’, which is represented by *M*_3_(*i*,* k*) = *M*_3_(*k*,* i*) = 0 for all *i* where 1 ≤ *i* ≤ *N*,* i* ≠ *k*.

It should be noted here that:

- There are no explicit ‘birth’ processes in this algorithm. Because our system is completely defined by the vertices and edges present and because the number of vertices remains constant, a birth process would simply be an addition of edges with a certain probability, which would essentially be indiscernible from our stepwise edge addition.
- The above algorithm is not strictly that of a random graph process in the mathematical sense. We will recall that the requirements for a random graph process are that each graph is a subgraph of the one after it and as we are incorporating a certain probability of taking edges away in the present model, this is purely a stochastic simulation and the death process will not be considered in our analytical model in the following section.

**Figure 8:**
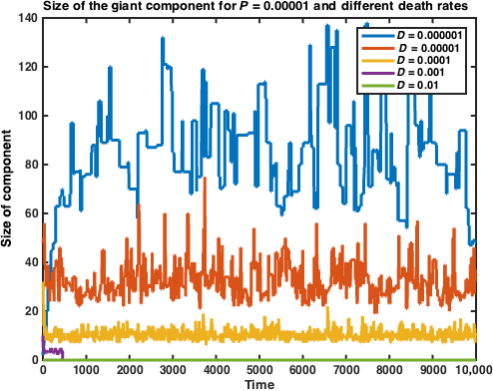
Size of the giant component vs. time in Model 3, for different values of the death rate *D*. Data represent average of *n* = 10 simulations. We can see that convergence does not occur within 10,000 timesteps and that for high death rates, the giant component does not have a chance to grow at all.

We run the above algorithm for an assembly of *N* = 1000 cells and can see that the imposed death rate prohibits convergence of the giant component at *t* = 10,000. These results are very interesting in light of our results from Models 1 and 2, and contextualizing them with experimental results suggests that death rates in bacterial colonies are not necessarily prohibitive to convergence of the giant connected component, although the development of the component itself is due to intercellular interaction. In our ‘Experimental Directions’ section, we discuss the implications of this finding, both for the understanding of the underlying population structure of the swarming population and for our understanding of the nature of cellular interactions during swarming (Fig. 8).

## Analytical results

Although the above treatment is good as a general model for understanding the dynamics of swarming and possible parameters affecting this process, it benefits us to extend this model to some measurable characteristic of the bacterial population so that it may be tested. In this section, we give analytical results for the probability of reaching a connected component of a certain size and of maximum degree 2 in a population of cells, given a certain interaction probability.

Experimentally, it is observed that swarming bacteria form both tendril‐like, uneven ring and concentric ring patterns. We hypothesize that tendril‐like and uneven ring shapes in swarming colonies can be attributed to localized clusters of cells reaching their threshold connected component sizes at different times, whereas concentric circles are formed through synchronicity in achievement of the connected component. In other words, the distribution in radii measured around the colony is related to the interaction probability of the cells. Let us denote the cellular density threshold *m*, where 1 ≤ *m* ≤ *n*. Bollobas 13 found that for the graph on the set of vertices *V*, almost every Erdos–Renyi random graph of this size contains a path of minimum length (1 − α(*c*))^*n*^, where α(*c*) is an exponentially decreasing function of *c*, a measure of the edge probability of the random graph. We can change some of the parameters for the model to make it fit our application, but it requires that we think about the assembly somewhat abstractly. We take the total number of cells in the entire assembly to be *N*, and represent it as a sum of clumps of cells, which we can take to be equal. Let us choose the number of elements in each clump to be such that within the context of the structure of the ring, they are ‘dense clumps’ of vertices, so that for a given clump, a connection that makes biological sense (*e.g*. an interaction through cell–cell adhesion proteins) can be made between any two vertices in the clump.

Let us say that within a given one of these clumps, the edges are chosen with probability *c*/*n*_*i*_. If we have *n*_1_ = *n*_2_ = *n*_3_ = *n*_*i*_, we can simplify this to *c*/*n*. If we take into account the threshold *m*, we have

(1) $$m\geq (1-e^{-c})n$$

Because each edge has an equal chance of appearing, we will also assume that there are equal weights attributed to travelling along an edge, and once you reach the threshold probability, it is only a question of a Markov chain reaching time to reach your result. In this case, each step in the Markov chain is a stage in the graph process, and so we have this time being proportional to the inverse of the probability of reaching this step:

(2) $$P={\left(\frac{c}{n}\right)}^{i}{\left(1-\frac{c}{n}\right)}^{v-i}$$

(3) $$T\;\;\infty \;\;{\left({\left(\frac{c}{n}\right)}^{i}{\left(1-\frac{c}{n}\right)}^{v-i}\right)}^{-1}$$

Alon and Chung 22 demonstrate the following: For every ε > 0 and every integer *m* ≥ 1, there is a graph *G*, which can be explicitly constructed, with $O\left(\frac{m}{\varepsilon }\right)$ vertices and maximum degree $O\left(\frac{1}{\varepsilon^{2}}\right)$ such that after deleting all but ε portion of its vertices *or* edges, the remaining graph still contains a path of length *m*.

The graph described above (after 0 < *x* < ε of the vertices have been removed) is essentially a random graph of the Erdos–Renyi model. To investigate the connections between these two graphs, we can draw a parallel between the $\frac{c}{n}$ in Eq. 2 and Eq. 3 and the ε in the second. From this, we have $\frac{c}{n}=\varepsilon$ and so we know that the graph in question has $\frac{mn}{c}$ vertices and maximum degree $\frac{n^{2}}{c^{2}}$. From Eq. 1, we have that

(4) $$\frac{m}{n}\geq 1-e^{-c}$$

(5) $$c\geq \ln\left(1-\frac{m}{n}\right)$$

The primary biological and mathematical question that is being asked in this study is that of randomness and probabilities within the biological network. We create a framework for understanding the architecture of an interacting assembly of cells here. For a given cell, the probability that it will participate in an interaction that falls within the threshold *m* that was defined earlier is

(6) $$P\left(t\right)\;\;\infty \;\;\left(\begin{array}{c} t\\ j \end{array}\right){\left(\frac{c}{n}\right)}^{i}{\left(1-\frac{c}{n}\right)}^{t-i}$$

where to make Eq. 6 physically realistic, $c\geq |\ln\left(1-\frac{m}{n}\right)|$. We have *i* = 1 since after a chain of cells (the giant component) has formed we are done. We simply want the probability of the Markov chain hitting the intended result within a certain threshold time *t* which we can take to be measured in discrete steps.

**Figure 9:**
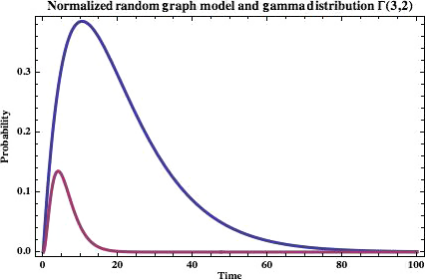
Our probability function for hitting a raft at a given time *t*. Graph shown is for *m *=* *6 and *n* = 10. The smaller graph shown is the gamma distribution Γ(3,2) for comparison.

We set *c* to be minimal, *i.e*. $c=|\ln\left(1-\frac{m}{n}\right)|$, so that we have now:

(7) $$P\left(t\right)\;\;\infty \;\;t\left(\frac{|\ln\left(1-\frac{m}{n}\right)|}{n}\right){\left(1-\frac{|\ln\left(1-\frac{m}{n}\right)|}{n}\right)}^{t-1}$$

which is the lower bound on *P* for a given *m, n* and *t*. Let us graph this result for 1 < *t* < 100, *m* = 6 and *n* = 10. We can see that it assumes the general form of a gamma (Γ) distribution (Fig. 9).

**Figure 10:**
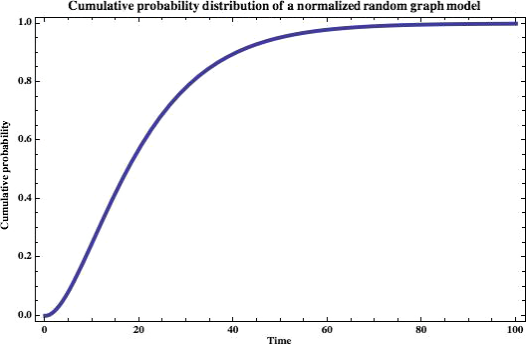
The cumulative probability distribution function for hitting a raft at a given time *t*. Graph shown is for *m *=* *6 and *n* = 10.

To observe asymptotic behaviour, we can represent our probability distribution function as a cumulative function (Fig. 10).

As expected, we can see that as the time is increased from *t* = 0, the probability of hitting the rafting threshold within that time increases as well, to a maximum. We can analytically find out where this maximum lies by taking the first derivative, which is

(8) $$P^{\prime }\left(t\right)\;\;\infty \;\;\frac{\left|\ln\left(1-\frac{m}{n}\right)\right|{\left(\frac{n-\left|\ln\left(1-\frac{m}{n}\right)\right|}{n}\right)}^{t-1}}{n}+\frac{t}{n}\left|\ln\left(1-\frac{m}{n}\right)\right|\ln\left(\frac{n-\left|\ln\left(1-\frac{m}{n}\right)\right|}{n}\right){\left(\frac{n-\left|\ln\left(1-\frac{m}{n}\right)\right|}{n}\right)}^{t-1}$$

and then setting this equal to 0, which yields

(9) $$t_{\max}=\frac{-1}{\ln\left(1-\frac{\left|\ln\left(1-\frac{m}{n}\right)\right|}{n}\right)}$$

As a coherence check, having a *n* value that is too large makes this value approach infinity, as we might expect. The function in Figure 9 resembles a gamma distribution, also shown. An interesting idea to present here is that we have not yet ruled out the possibility of our threshold being a function of *t* we can still see if this is the case. For some proportionality constant *A*, we represent our model in the form

(10) $$A\frac{t^{k-1}e^{\frac{-t}{\theta }}}{\theta^{k}\left(k-1\right)!}=t\left(C\right){\left(1-C\right)}^{t-1}$$

where

(11) $$C=\frac{\left|\ln\left(1-\frac{m}{n}\right)\right|}{n}$$

Performing a series expansion of the two sides of the equation and matching the first‐order terms (terms that are approximately proportional to *t*), we have

(12) $$-\frac{AC}{C-1}=\frac{\theta^{-k-2}}{2(k-1)!}$$

We solve for *C* to obtain:

(13) $$C\approx \frac{t^{k}}{2A\left(k-1\right)!\theta^{k+2}+t^{k}}$$

and using our definition for *C*, we have:

(14) $$\frac{-\ln\left(1-\frac{m}{n}\right)}{n}\approx \frac{t^{k}}{2A\left(k-1\right)!\theta^{k+2}+t^{k}}$$

(15) $$m\left(t\right)=n\left(e^{\frac{nt^{k}}{2A\left(k-1\right)!\theta^{k+2}+t^{k}}}-1\right)$$

When *k* << 1, we approximate *t* ^*k*^ ≈ 1:

(16) $$m\left(t\right)=n\left(e^{\frac{n}{2A\left(k-1\right)!\theta^{k+2}+1}}-1\right)$$

As we can see, *m* is in fact approximately a constant independent of *t* for small *k* and can be related to the values generated for gamma fits of experimental data by the above equation.

## Experimental directions

Having formulated a graph theoretical model for the dynamics of swarming bacteria, we turn our sights to the experimental domain. There are several ways in which to expand upon this model, which are briefly outlined in this section.

### Normalizing our analytical model

Although this model is enlightening in its explanation of trends in various swarming phenomena, it lacks very much practical application without at least a general understanding of the values that *m* and *n* can take, which can in turn help us understand our scaling factor *A*. With reference to papers seeking to find the density threshold of *P. mirabilis* swarming 19, 20, we can see that the ratio of swarmers to swimmers in a consolidation layer of *P. mirabilis* strain PM23 that is ready to swarm is roughly 1:1.5, which corresponds to $\frac{m}{n}=0.6$. We plug in *m* = 60, *n* = 100 and our experimentally derived values for *k*, θ to get *A* ≈ −3.06 × 10^−6^.

It is important to note that this is only an example of a value that *A* can take. Swarming behaviour is different from strain to strain of *P. mirabilis*, and so the same experiments on density would have to be performed to properly normalize the model for different *P. mirabilis* strains.

## Discussion

In the current study, we explore the use of random graph processes to find analytical times for attaining a threshold giant ‘chain’ component within a spatial cellular network, in which edges signify interactions between cells. We demonstrate a possible use for this model in interpreting density‐dependent biological data and show that under a small gamma distribution parameter *k*, the interaction threshold for a certain population is independent of time and can be calculated from experimental data.

The inspiration for this model, as discussed previously, stems from the phase change threshold of connectivity within a random graph as described by random cluster theory in condensed matter physics. In the aforementioned model, an energetic consideration can be made, since we can simply assign an energy to different types of interactions and compute the partition function of the entire graph to obtain a free energy measurement for the whole graph. The free energy measurement of massive particles such as cells in media cannot be generalized in this way to a high degree of accuracy; however, the emergence of certain density‐dependent behaviours over time can be used to collect data to test the accuracy of the model.

It should also be noted that the phenomenon studied is similar to the emergence of the ‘giant component’ in a random graph process; however, this is a specific example of the evolution of a giant component in the graph. The evolution of a giant component in a random graph process occurs through the existence of a variable distribution of degrees of connectivity throughout the vertices in the connected component. Here, we attempt to apply this formalism to biological systems, in which cells and organisms have an upper bound on the number of connections they can make; a good example of this is through cell–cell adhesion in the cells of the stomach lining, which occurs on the basolateral sides of cells rather than on the apical side facing the lumen of the stomach. Swarming is also an example of this process in that there is a definite upper limit on the degree of any vertex in a spatial population graph of bacterial cells. For this reason, the current study examines ‘one‐to‐one’ chains of interacting cells.

## Funding

This work was supported by a summer grant from the Origins of Life Initiative awarded to SGJ.

## Conflicts of Interest

The author has no competing interests to declare.
